# 3D visualization model construction based on generative adversarial networks

**DOI:** 10.7717/peerj-cs.768

**Published:** 2022-03-29

**Authors:** Xiaojuan Liu, Shangbo Zhou, Sheng Wu, Duo Tan, Rui Yao

**Affiliations:** 1 College of Computer Science, Chongqing University, Chongqing, China; 2 Key Laboratory of Dependable Service Computing in Cyber Physical Society, Ministry of Education, Chongqing University, Chongqing, China; 3 College of Computer and Information Science, SouthWest University, Chongqing, China

**Keywords:** 3D visualization model, Neural network, Generation adversarial network, Precision components

## Abstract

The development of computer vision technology is rapid, which supports the automatic quality control of precision components efficiently and reliably. This paper focuses on the application of computer vision technology in manufacturing quality control. A new deep learning algorithm is presented, Multi-angle projective Generative Adversarial Networks (MapGANs), to automatically generate 3D visualization models of products and components. The generated 3D visualization models can intuitively and accurately display the product parameters and indicators. Based on these indicators, our model can accurately determine whether the product meets the standard. The working principle of the MapGANs algorithm is to automatically infer the basic three-dimensional shape distribution through the product’s projection module, while using multiple angles and multiple views to improve the fineness and accuracy of the three-dimensional visualization model. The experimental results prove that MapGANs can effectively reconstruct two-dimensional images into three-dimensional visualization models, and meanwhile accurately predict whether the quality of the product meets the standard.

## Introduction

The globalization of information and industry has made a huge impact on the traditional manufacturing industry. This has forced the traditional manufacturing industry to constantly innovate and create new technologies and methods to meet the needs of the times and the market. On the one hand, the traditional centralized manufacturing system can no longer meet the needs of the market. On the other hand, with the continuous development of Internet technology, a large number of emerging concepts and methods have been proposed to improve product design, distribution and production, which include distributed collaborative design and production, product visualization, virtual reality and augmented reality (Zhang, Shen & Ghenniwa, 2004). All of these technologies cannot be achieved without information sharing and visualization.

Different from other fields, manufacturing encompasses a complete chain: marketing, supply, product design and production, production floor control, process planning, quality control and business management. Over the past 20 years, manufacturing has gradually begun to integrate with market analysis, product design and production to achieve distributed management and control. In this process, it becomes critical to monitor and control product quality efficiently and accurately. Traditional production lines on the shop floor are monitored and managed mainly through human labor. This method is not only labor-intensive, but usually only detects some rough quality problems, such as the weight or length of the product. Although many methods have been proposed to refine product parameters and metrics, most systems are unable to effectively monitor precision components.

With the continuous development of computer technology, especially computer vision, it has become possible to monitor the quality of precision components automatically. Cameras mounted on machines can take pictures of products from different angles and then detect subtle visual clues about quality problems through sophisticated computer vision algorithms in the background. Numerous studies and case studies have proven that machine learning, especially deep learning, can dramatically improve quality control tasks in large assembly lines. According to FORBES, analytics and machine learning-driven process and quality optimization is expected to grow 35%, while process visualization and automation is expected to grow 34%.

In this paper, we will discuss how to realize the application of computer vision technology in manufacturing quality control. First, we will describe how to construct a 3D visualization model from multi-angle photos taken by a camera. The generated 3D visualization model can be used to assist and control the production of high quality products from many different perspectives. Secondly, all the generated 3D visualization models will be transmitted to the information sharing platform. The technicians can clearly and intuitively observe the status and quality of the product through the shared platform, so that they can adjust the production line in a timely and effective manner. At the same time, our method can also automatically determine whether the product meets the standards based on the size, shape and other parameters of the 3D visualization model. Once the product’s compliance rate is too low, the system will automatically issue a warning to remind technicians to check the equipment and production process. Compared to 2D images, the 3D visualization model has a huge advantage in that it can accurately detect the product’s parameters and indicators without being disturbed by factors such as the angle of light taken in the 2D view. This was also confirmed in later experiments, where the 3D visualization model was able to achieve a much higher accuracy rate of quality inspection than the 2D view.

To generate 3D visualization models, a novel Multi-angle projective Generative Adversarial Networks (MapGANs) is proposed in this paper. It can train a deep network to build 3D visualization models by using multi-angle 2D images and projections. The main advantages of the model include: (1) The addition of the projection module allows us to infer the basic three-dimensional shape distribution without using any three-dimensional viewpoint information in the learning phase. (2) The use of multiple angles and multiple views can improve the fineness and accuracy of the 3D visualization model. (3) For some more complex parts, the camera must have an occlusion problem. The algorithm proposed in this paper can combine several 2D views from different angles to automatically associate and fill in the residuals due to occlusion and lighting, etc., so as to generate an accurate 3D visualization model.

The experimental results also show that MapGANs algorithm can reconstruct 2D images into 3D visualization models very accurately, and at the same time automatically determine the product qualification rate based on different product parameters on the 3D model.

### Current research

A large amount of insightful research has recently emerged in the area of 3D visual modeling. Much of this work is based on an assembly approach to construct deformable component assembly models (Chaudhuri et al., 2011; Kossaifi et al., 2019; Goodfellow et al., 2020). These methods are limited to specific types of shapes with small variations, and surface correspondence is one of the key issues in such methods. Typically, such part annotation-based modeling methods are cumbersome and expensive, so assembly-based modeling is difficult to be applied to real-life applications. Another part of the work is mainly based on smooth interpolation or extrapolation to reconstruct the surface of a damaged scan input. These methods can only resolve small missing holes or defects (Shalom et al., 2010; Yu et al., 2020; Zhu et al., 2018). Template-based approaches can handle larger spatial disruptions, but they are limited by the quality of available templates and often do not provide different semantic interpretations of the reconfiguration (Shen et al., 2012; Ji et al., 2019).

The powerful generative power of deep learning models has enabled researchers to build models for 3D visualization, most notably Deep Belief Network (DBN) to generate handwritten numbers and ShapeBM to generate the shape of a horse. These models are able to effectively capture changes in picture details. For deep learning in 2.5 dimensions, the literature (Socher et al., 2012; Pavlakos et al., 2018) builds discriminative convolutional neural networks to model images and depth graphs. Although their algorithms are applicable to depth graphs, they use the depth of the graph as an additional 2D channel, however they are unable to generate 3D models.

Optimization-based models estimate a priori geometric, material, and light information by minimizing reconstruction errors during rendering (Barron & Malik, 2015). Recognition-based methods have been widely used to estimate outdoor scenes, indoor environments and object geometries. Recently, many methods generate 3D visualization views by means of convolutional networks that have been trained with the attributes and parameters of a given 3D object. Most of these recognition-based methods are trained in a fully supervised manner and require 3D data or views of the same object from multiple views during the training process (Yan et al., 2016).

The current 3D reconstruction method based on deep learning is to compare the predicted shape with the real 3D model, which used the loss function minimization to make the predicted shape more and more accurate. Considering the diversity of models, it’s hard to learn useful details during training. Due to the generative adversarial network has the ability of learning and image generation, we can learn the features through the confrontation training between the generator and the discriminator in the network. This method provides a new idea for 3D image construction.

Our work is based on Generative Adversarial Networks (GAN). Generative adversarial networks have been used in many different domains to generate views. Recently, the literature (Wu et al., 2016) learned generative models for 3D visualized shapes by using a variant of adversarial networks equipped with 3D convolutions. However, the models were trained using aligned 3D shape data. However, the models are trained using aligned 3D shape data. Our work aims to address the challenge of learning 3D visualization models from 2D images. Recently, the literature (Choy et al., 2016) used projections of object instances to form one or more images and output reconstructed images of the objects in the form of a 3D occupancy grid. Unlike most previous works, their network does not require any image annotation or object class labeling for training or testing. Our method also uses the projection of object instances to help reconstruct the images. Meanwhile, we also add 2D images taken from multiple angles to train a more effective deep network to build 3D visualization models.

### Generative adversarial networks

Generative Adversarial Networks (GAN) is a kind of machine learning system invented by Ian Goodfellow and colleagues in 2014 (Goodfellow et al., 2014). Two neural networks compete with each other in a game (usually but not always in the form of a zero-sum game, in terms of game theory). Given a training set, the technique will learn to generate new data with the same statistics as the training set. For example, a photo-trained GAN can generate new photos that appear real, at least on the surface, to a human observer with many realistic features. Although originally proposed as a generative model for unsupervised learning, GAN has also been shown to be useful for semi-supervised learning (Salimans et al., 2016), Completely supervised learning (Isola et al., 2017), and reinforcement learning (Ho & Ermon, 2016).

The GAN model architecture involves two sub-models: the generator model, which is used to generate new reasonable examples from the problem domain, and the discriminator, which is used to discriminate real or faked examples against the generator-generated examples (Mohamed et al., 2018; Cao et al., 2018). The generator model takes as input a fixed-length random vector and generates samples in the domain. The vector is randomly drawn from a Gaussian distribution and the vector is used to propagate the generation process (Sun et al., 2018). After training, the points in this multidimensional vector space will correspond to the points in the problem domain, resulting in a compressed representation of the data distribution. The discriminator model takes the examples in the domain as input (real or generated) and discriminates between generating real or forged binary class labels. The real examples are generally derived from the training dataset. The generated examples are output by the generator model. In fact, a discriminator is a normal classification model (Fan, Su & Guibas, 2017; Kar et al., 2015).

### 3D scene construction based on generative adversarial networks

**Figure 1:**
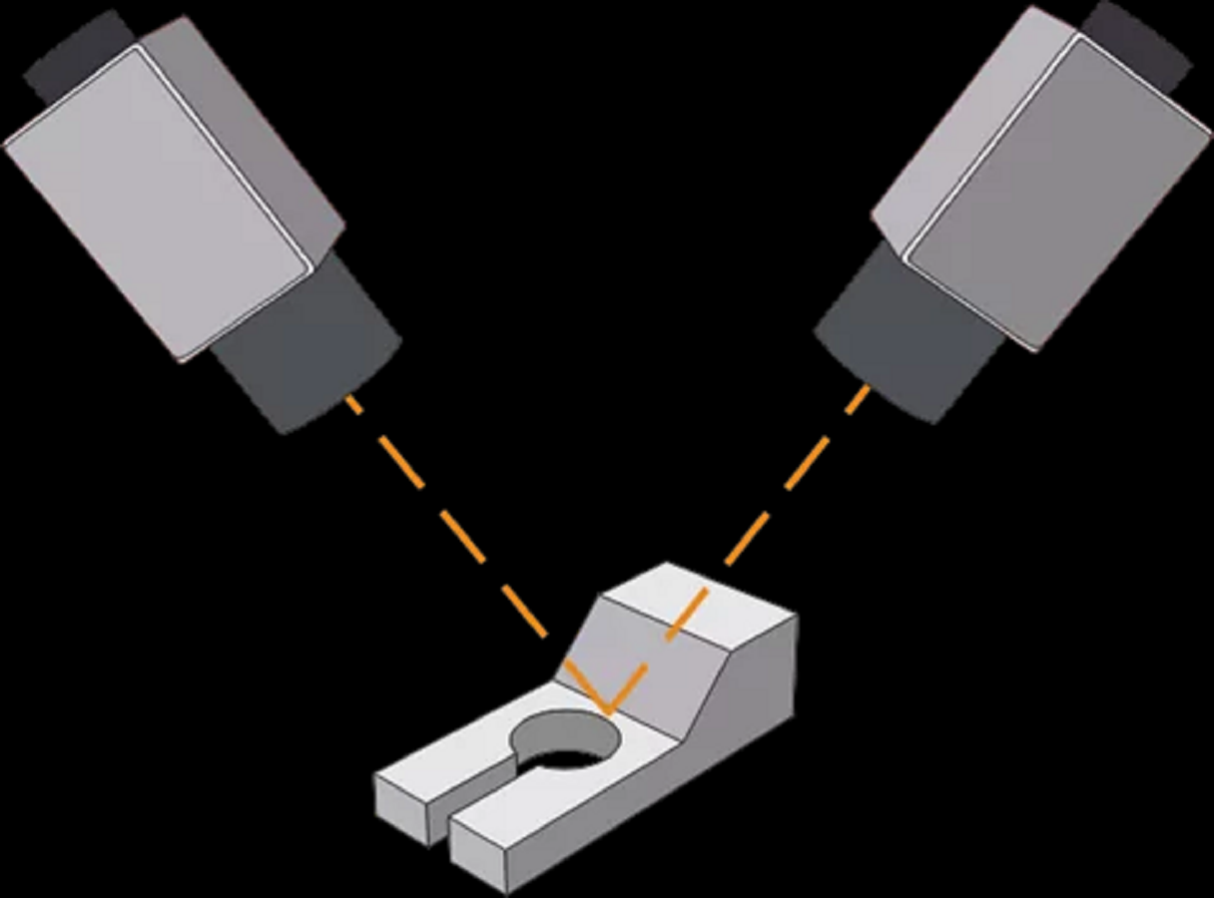
Example of a camera on a production line.

In order to generate 3D visualization models, multiple cameras need to be installed on the production line to take pictures from different angles and different locations. Our approach will generate 3D visualization models based on the multiple photos taken by deep learning neural networks. Figure 1 shows an example of a camera installed on a production line. In a real-world environment, we will install multiple cameras to capture from different angles and different positions. The multi-angle shots help our algorithm to better identify object size, shape and subtle visual cues. Meanwhile, this also enhances the robustness of the algorithm for different environments, including different light brightness and possible occlusions.

Our approach is based on the GAN architecture proposed by Goodfellow et al. (2020). The model consists of two parts: the generator and the discriminator. In order to learn the distribution *p*_g_ of the generator G from the data x,First, it is necessary to define a priori noise variable z and its distribution *p*_*z*_. Then the GAN represents the mapping from the noise space to the data space as $\mathrm{G} \left( \mathrm{z},{\mathrm{\theta }}_{\mathrm{g}} \right) $, where G is a differentiable function of the neural network with parameter. θ_g_. The discriminator $\mathrm{D} \left( \mathrm{x},{\mathrm{\theta }}_{\mathrm{d}} \right) $ is also defined with the parameter *θ*_d_ and the output of $\mathrm{D} \left( \mathrm{x} \right) $ is a single scalar. The parameter x of $\mathrm{D} \left( \mathrm{x} \right) $ indicates that the probability is from the data and not from the generator G. The GAN will train the discriminator D to maximize the probability of providing correct labels for the training data and the samples generated from the generator G, while minimizing $\log \left( 1-\mathrm{D} \left( \mathrm{G} \left( \mathrm{z} \right) \right) \right) $.

Therefore, the objective function of GAN can be expressed a

(1) \begin{eqnarray*}mi{n}_{G}ma{x}_{D}\mathrm{V } \left( D,G \right) ={\mathrm{E}}_{\mathrm{x}\sim {\mathrm{p}}_{\mathrm{d}} \left( \mathrm{x} \right) } \left[ \log \nolimits \mathrm{D} \left( \mathrm{x} \right) \right] +{\mathrm{E}}_{\mathrm{z}\sim {\mathrm{p}}_{\mathrm{z}} \left( \mathrm{z} \right) } \left[ \log \nolimits (1-\mathrm{D}(\mathrm{G}(\mathrm{z}))) \right] \end{eqnarray*}

$\log \mathrm{D} \left( \mathrm{x} \right) $ is the cross entropy between ${ \left[ 1,0 \right] }^{T}$ and ${ \left[ D \left( x \right) ,1-D \left( x \right) \right] }^{T}$ Similarly, $\log (1-\mathrm{D}(\mathrm{G}(\mathrm{z})))\log \mathrm{D} \left( \mathrm{x} \right) $ is the cross entropy between ${ \left[ 0,1 \right] }^{T}$ and ${ \left[ D \left( G \left( z \right) \right) ,1-D \left( G \left( z \right) \right) \right] }^{T}$. For a fixed G, the optimal discriminator D is

(2) \begin{eqnarray*}{D}_{G}^{\ast } \left( x \right) = \frac{{\mathrm{p}}_{\mathrm{d}} \left( \mathrm{x} \right) }{{\mathrm{p}}_{\mathrm{d}} \left( \mathrm{x} \right) +{\mathrm{p}}_{\mathrm{g}} \left( \mathrm{x} \right) } \end{eqnarray*}

Therefore, Eq. (1) can be expressed as

$ \mathrm{C} \left( \mathrm{G} \right) =ma{x}_{D}\mathrm{V } \left( D,G \right) $

\begin{eqnarray*} & ={\mathrm{E}}_{\mathrm{x}\sim {\mathrm{p}}_{\mathrm{d}}} \left[ \log \nolimits {\mathrm{D}}_{\mathrm{G}}^{\mathrm{\ast }} \left( \mathrm{x} \right) \right] +{\mathrm{E}}_{\mathrm{ z}\sim {\mathrm{p}}_{\mathrm{z}}} \end{eqnarray*}

\begin{eqnarray*} & ={\mathrm{E}}_{\mathrm{x}\sim {\mathrm{p}}_{\mathrm{d}}} \left[ \log \nolimits {\mathrm{D}}_{\mathrm{G}}^{\mathrm{\ast }} \left( \mathrm{x} \right) \right] {\mathrm{E}}_{\mathrm{ z}\sim {\mathrm{p}}_{\mathrm{g}}} \left[ \log \nolimits (1-{\mathrm{D}}_{\mathrm{G}}^{\mathrm{\ast }}(\mathrm{x})) \right] \end{eqnarray*}

(3) \begin{eqnarray*}={\mathrm{E}}_{\mathrm{x}\sim {\mathrm{p}}_{\text{data}}} \left[ \log \nolimits \frac{{\mathrm{p}}_{\mathrm{d}} \left( \mathrm{x} \right) }{ \frac{1}{2} \left( {\mathrm{p}}_{\mathrm{d}} \left( \mathrm{x} \right) +{\mathrm{p}}_{\mathrm{g}} \left( \mathrm{x} \right) \right) } \right] +{\mathrm{E}}_{\mathrm{x}\sim {\mathrm{p}}_{\mathrm{g}}} \left[ \log \nolimits \frac{{\mathrm{p}}_{\mathrm{g}} \left( \mathrm{x} \right) }{ \frac{1}{2} \left( {\mathrm{p}}_{\mathrm{d}} \left( \mathrm{x} \right) +{\mathrm{p}}_{\mathrm{g}} \left( \mathrm{x} \right) \right) } \right] -2\log \nolimits 2\end{eqnarray*}

Between two given probability distributions $\mathrm{p} \left( \mathrm{x} \right) $ and $\mathrm{q} \left( \mathrm{x} \right) $, The KullbackLeibler (KL) scatter and the Jensen–Shannon (JS) scatter can be defined as

(4) \begin{eqnarray*}\mathrm{KL}(\mathrm{p}{|}{|}\mathrm{q})=\int \nolimits \mathrm{p} \left( \mathrm{x} \right) \log \nolimits \frac{\mathrm{p} \left( \mathrm{x} \right) }{\mathrm{q} \left( \mathrm{x} \right) } \mathrm{dx}\end{eqnarray*}

(5) \begin{eqnarray*}\mathrm{JS}(\mathrm{p}{|}{|}\mathrm{q})= \frac{1}{2} \mathrm{KL}(\mathrm{p}{|}{|} \frac{\mathrm{p}+\mathrm{q}}{2} )+ \frac{1}{2} \mathrm{KL}(\mathrm{q}{|}{|} \frac{\mathrm{p}+\mathrm{q}}{2} )\end{eqnarray*}

Substituting Eqs. (4) and (5) into (3), we can get

(6) \begin{eqnarray*}\mathrm{C}(\mathrm{G})=\mathrm{KL}({\mathrm{p}}_{\text{data}}{|}{|} \frac{{\mathrm{p}}_{\mathrm{d}}+{\mathrm{p}}_{\mathrm{g}}}{2} )+\mathrm{KL}({\mathrm{p}}_{\mathrm{g}}{|}{|} \frac{{\mathrm{p}}_{\mathrm{d}}+{\mathrm{p}}_{\mathrm{g}}}{2} )-2\log \nolimits 2=2\mathrm{JS}({\mathrm{p}}_{\mathrm{d}}{|}{|}{\mathrm{p}}_{\mathrm{g}})-2\log \nolimits 2\end{eqnarray*}

Therefore, the objective function of GAN is related to both KL scatter and JS scatter.

### Multi-angle projection of generative adversarial networks

In this paper, a novel multi-angle projection generative adversarial network is proposed. Unlike traditional generative adversarial networks, the network proposed in this paper will introduce pictures taken from multiple different angles to enrich the input data of the model. This method will effectively weaken or eliminate the error and noise brought by a single data, and can present the real shape of the object more clearly.

**Figure 2:**
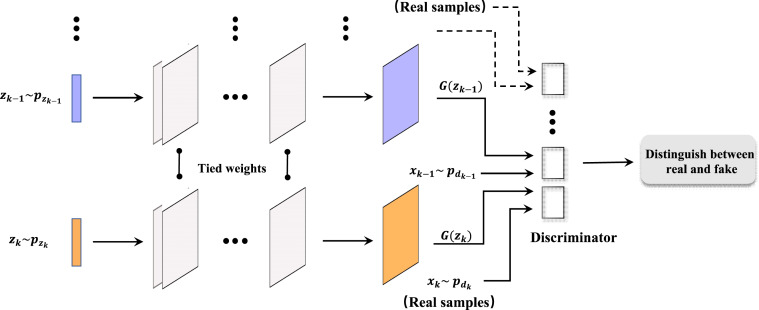
Architecture of MapGAN model.

Figure 2 illustrates the architecture of a multi-angle projection generative adversarial network (MapGAN). Similar to the traditional generative adversarial network, the architecture also consists of a generator and a discriminator. The difference is that it introduces several different input data *x*_1_,  *x*_2_, …,  *x*_*k*_, and the corresponding prior probabilities to generate *z*_1_,  *z*_2_, …,  *z*_*k*_. And therefore, multiple discriminators are added to separately identify whether the examples are real or fake. Also, we see that the parameters are bound between each set of generators and discriminators, with multiple sets of different training data in order to obtain the optimal parameter values.

In fact, the objective functions of each group of generators and discriminators remain the same. The objective function of the i-th group can be expressed as

(7) \begin{eqnarray*}{\mathrm{E}}_{{\mathrm{x}}_{i}\sim {\mathrm{p}}_{{\mathrm{d}}_{i}}} \left[ \log \nolimits {\mathrm{D}}_{k+1} \left( \mathrm{x};{\theta }_{{d}_{i}} \right) \right] +{\mathrm{E}}_{{\mathrm{z}}_{i}\sim {\mathrm{p}}_{{\mathrm{z}}_{i}}} \left[ \log \nolimits (1-{\mathrm{D}}_{k+1}(\mathrm{G} \left( {\mathrm{z}}_{i};{\theta }_{g}^{i} \right) ;{\theta }_{{d}_{i}})) \right] \end{eqnarray*}

To update the parameters, the gradient of each generator can be simply expressed as ${\nabla }_{{\theta }_{g}^{i}}\log (1-\mathrm{D}(\mathrm{G} \left( {\mathrm{z}}_{i};{\theta }_{g}^{i} \right) ;{\theta }_{{d}_{i}}))$. It is worth noting that since all parameters are bound, the parameters of all these generators will be updated simultaneously. For the discriminator, given *x* ∼ *p* (real or forged) and the corresponding *δ*, the gradient can be expressed as ${\nabla }_{{d}_{i}}\log {\mathrm{D}}_{j} \left( \mathrm{x},{\theta }_{{d}_{i}} \right) $. where ${\mathrm{D}}_{j} \left( \mathrm{x},{\theta }_{{d}_{i}} \right) $ is the j-th coefficient of $D \left( \mathrm{x},{\theta }_{{d}_{i}} \right) $ for $\delta \left( j \right) =1$. Therefore, using this approach requires very small modifications to the standard GAN optimization algorithm and can be easily used with different variants of GAN.

### Theories analysis

Theorem 1 shows that the above objective function (Eq. 4) can actually allow the generators to form a mixture model, where each generator represents a mixture component. And when ${p}_{d}= \frac{1}{k} {\mathop{\sum }\nolimits }_{i=1}^{k}{p}_{{g}_{i}}$ , the global optimal solution is $- \left( k+1 \right) \log \left( k+1 \right) +k\log k$. Note that when *k* =1, that is, when only one generator is included, the model will obtain exactly the same Jensen–Shannon divergence as in Socher et al. (2012), and the optimal value is −log4.

**Theorem 1**

Given an optimal discriminator, the objective function of the training generator can be reduced to minimizing

(8) \begin{eqnarray*}KL({p}_{}d(x){|}{|}{p}_{avg}(x)+kKL \left( \frac{1}{k} \sum _{i=1}^{k}{p}_{}{g}_{}i \left( x \right) {|}{|}{p}_{}avg \left( x \right) \right) )- \left( k+1 \right) \log \nolimits \left( k+1 \right) +k\log \nolimits k\end{eqnarray*}

where ${p}_{avg} \left( x \right) = \frac{{p}_{}d \left( x \right) +{\sum }_{}{i=1}^{k}{p}_{}{g}_{}i \left( x \right) }{k+1} $. When ${p}_{d}= \frac{1}{k} {\mathop{\sum }\nolimits }_{i=1}^{k}{p}_{{g}_{i}}$, the objective function (Eq. 8) will obtain the global minimum value $- \left( k+1 \right) \log \left( k+1 \right) +k\log k$.

**Proof:**

The joint goal of all generators is to minimize

(9) \begin{eqnarray*}{\mathrm{E}}_{\mathrm{x}\sim {\mathrm{p}}_{\mathrm{d}}} \left[ \log \nolimits {\mathrm{D}}_{k+1} \left( \mathrm{x} \right) \right] +\sum _{i=1}^{k}{\mathrm{E}}_{\mathrm{ x}\sim {\mathrm{p}}_{{g}_{i}}} \left[ \log \nolimits (1-{\mathrm{D}}_{k+1}(\mathrm{x})) \right] \end{eqnarray*}

Using Corollary 1, we replace the optimal discriminator with the above equation and obtain

\begin{eqnarray*}{\mathrm{E}}_{\mathrm{x}\sim {\mathrm{p}}_{\mathrm{d}}}\log \nolimits \frac{{p}_{d} \left( x \right) }{{p}_{d} \left( x \right) +\sum _{i=1}^{k}{p}_{{g}_{i}} \left( x \right) } +\sum _{i=1}^{k}{\mathrm{E}}_{\mathrm{ x}\sim {\mathrm{p}}_{{g}_{i}}}\log \nolimits \frac{\sum _{i=1}^{k}{p}_{{g}_{i}} \left( x \right) }{{p}_{d} \left( x \right) +\sum _{i=1}^{k}{p}_{{g}_{i}} \left( x \right) } \end{eqnarray*}

(10) \begin{eqnarray*}={\mathrm{E}}_{\mathrm{x}\sim {\mathrm{p}}_{\mathrm{d}}}\log \nolimits \frac{{p}_{d} \left( x \right) }{{p}_{avg} \left( x \right) } +\mathrm{k}{\mathrm{E}}_{\mathrm{x}\sim {\mathrm{p}}_{\mathrm{g}}}\log \nolimits \frac{{\mathrm{p}}_{\mathrm{g}} \left( x \right) }{{p}_{avg} \left( x \right) } - \left( k+1 \right) \log \nolimits \left( k+1 \right) +k\log \nolimits k\end{eqnarray*}

Among, that is ${p}_{g}= \frac{{\mathop{\sum }\nolimits }_{i=1}^{k}{p}_{{g}_{i}}}{k} ,{p}_{avg} \left( x \right) = \frac{{p}_{d} \left( x \right) +{\mathop{\sum }\nolimits }_{i=1}^{k}{p}_{{g}_{i}} \left( x \right) }{k+1} $. Bringing Eqs. (4) into (7), we can see that Eq. (7) is the same as Eq. (5). Meanwhile, when ${p}_{d}= \frac{{\mathop{\sum }\nolimits }_{i=1}^{k}{p}_{{g}_{i}}}{k} $, the KL term will be 0 and we will get the global minimum.

**Corollary 1**

For a given generator, the optimal distribution obtained from the discriminator is

(11) \begin{eqnarray*}{D}_{k+1} \left( x \right) = \frac{{p}_{d} \left( x \right) }{{p}_{d} \left( x \right) +\sum _{i=1}^{k}{p}_{{g}_{i}} \left( x \right) } \end{eqnarray*}

(12) \begin{eqnarray*}{D}_{i} \left( x \right) = \frac{{p}_{{g}_{i}} \left( x \right) }{{p}_{d} \left( x \right) +\sum _{i=1}^{k}{p}_{{g}_{i}} \left( x \right) } ,\forall i \left\{ 1,\ldots ,k \right\} \end{eqnarray*}

where ${D}_{i} \left( x \right) $ refers to the i-th coefficient of $D \left( x;{\theta }_{d} \right) $, *p*_*d*_ is the distribution of the real data, and *p*_*g*_*i*__ is the distribution generated from the i-th generator.

**Proof:**

For a given generator, the discriminator’s objective function is to maximize

(13) \begin{eqnarray*}{\mathrm{E}}_{\mathrm{x}\sim {\mathrm{p}}_{\mathrm{d}}}\log \nolimits {\mathrm{D}}_{k+1} \left( \mathrm{x} \right) +\sum _{i=1}^{k}{\mathrm{E}}_{{x}_{i}\sim {\mathrm{p}}_{{p}_{{g}_{i}}}}\log \nolimits {D}_{i} \left( {x}_{i} \right) \end{eqnarray*}

Among, where ${\mathop{\sum }\nolimits }_{i=1}^{k}\log {D}_{i} \left( {x}_{i} \right) =1$ and ${D}_{i} \left( {x}_{i} \right) \in \left[ 0,1 \right] ,\forall i$. Equation (10) can be expressed as

(14) \begin{eqnarray*}\int \nolimits {p}_{d} \left( x \right) \log \nolimits {\mathrm{D}}_{k+1} \left( \mathrm{x} \right) dx+\sum _{i=1}^{k}\int \nolimits {p}_{{g}_{i}} \left( x \right) \log \nolimits {\mathrm{D}}_{i} \left( \mathrm{x} \right) dx=\int \nolimits \sum _{i=1}^{k}{p}_{i} \left( x \right) \log \nolimits {\mathrm{D}}_{i} \left( \mathrm{x} \right) dx\end{eqnarray*}

where ${p}_{k+1} \left( x \right) :={p}_{d} \left( x \right) ,{p}_{i} \left( x \right) :={p}_{{g}_{i}} \left( x \right) ,\forall i \left\{ 1,\ldots ,k \right\} $, and $Supp \left( p \right) ={\mathop{\bigcup }\nolimits }_{i=1}^{k}Supp \left( {p}_{{g}_{i}} \right) \cup Supp \left( {p}_{d} \right) $. Thus, given an x, the optimal objective function can be defined as Eq. (11), where the constraints can be obtained by the following property1.

**Property 1**

Given $\mathbi{y}= \left( {y}_{1},\ldots ,{y}_{n} \right) ,{y}_{i}\geq 0$,and *a*_*i*_ ∈ ℝ,when ${y}_{i}\ast = \frac{{a}_{i}}{{\mathop{\sum }\nolimits }_{i=1}^{n}{a}_{i}} ,\forall i$ ,

(15) \begin{eqnarray*}\max _{\mathbi{y}}\sum _{i=1}^{k}{a}_{i}log{y}_{i},s.t.\sum _{i}^{n}{y}_{i}=1.\end{eqnarray*}

**Proof:**

Transformation of the objective function into a Lagrangian function

(16) \begin{eqnarray*}L \left( \mathbi{y},\lambda \right) =\sum _{i=1}^{k}{a}_{i}log{y}_{i}+\lambda (\sum _{i=1}^{n}{y}_{i}-1).\end{eqnarray*}

Partially differentiate *y*_*i*_ and *λ* respectively, and get

(17) \begin{eqnarray*} \frac{{a}_{i}}{{y}_{i}} +\lambda =0,\sum _{i=1}^{n}{y}_{i}-1=0.\end{eqnarray*}

Solve the equation to get:

(18) \begin{eqnarray*}{y}_{i}\ast = \frac{{a}_{i}}{\sum _{i=1}^{n}{a}_{i}} .\end{eqnarray*}

### 3D reconstruction based on multi-angle projective generative adversarial networks

The initial input of this process is object images taken from different angles, the algorithm uses the training set for adversarial learning. Each iteration training uses the results generated by a set of generators to make multiple judgments. The generative adversarial networks use data from training sets to learn, each learning session produces an output. The parameters between each set of generators and discriminators are bound, therefore, the goal of training is to obtain the optimal parameter through multiple groups of different training data. Thus the convergence of the model is achieved and the 3D reconstruction of the object is realized.


 
_______________________ 
Algorithm 1 3D reconstruction based on Multi-angle projective Generative Ad- 
versarial Networks 
Input:A multi-angle image of an object 2.Output:The generated 3D model 3.for 
epochs do:  4.  for iterations do 5.  Group training for generator discriminator: 
6.  Generator ←random variable z 7.  Optimizing discriminator D 8.  D∗G (x) = 
    pd(x)_____ 
pd(x)+pg(x) 
9.  The discriminator constructed is used to judge the output of the genera- 
tor 10.  The constraints of each set of generators and discriminators are cal- 
culated 11 C(G)  =  KL(pdata||pd+pg 
    2    ) + KL(pg||pd+pg 
    2    ) − 2log 2 12.   end for 
13.   In  each  group,  the  discriminator  and  generator  are  spliced  to  form  a 
global GAN and an epoch is trained.  The combination requirements are as 
follows 14.  Exi∼pd 
i [log Dk+1 (x;θdi)] + Ezi∼pz 
i [ 
    log(1 − Dk+1(G( 
 zi;θig) 
     ;θdi))] 
15.   for  iterations  do:  16.   Training  the  global  generation  adversarial  net- 
work:  17.   Generate 3D reconstruction model:  18.   After step 14,  a mixed 
model is formed by using group generator and discriminator,a global gener- 
ation network is generated,pd =  1 
k ∑k 
       i=1 pgi,The optimal solution is searched 
min(KL(pd(x)||pavg (x)+kKL( 
1 
k ∑k 
        i=1 pgi (x)||pavg (x)) 
                 −(k + 1)log (k + 1)+ 
k log k)) 19.  end for 20.  The global discriminator and generator are combined 
to form a global generation adversarial network, then an epoch is trained.  21. 
end for______________________________________________________________________________________________    


### Experiment and analysis

### Data collection

In order to build a three-dimensional visualization model, multiple cameras need to be installed on the workshop assembly line. The camera will take product photos from different angles and positions. In this experiment, we installed 8 cameras on the shop floor assembly line, all of which were installed based on the years of working experience of professionals. This ensures that each camera can shoot from the best position, while the angle of the camera is adjusted according to the characteristics of different parts. The eight cameras will take pictures of each manufactured product and part, and each picture will be numbered according to the time it was taken and the camera number, so that it is easy to classify which pictures belong to the same product. All photos taken will be uploaded to the server *via* the shop floor’s wireless or wired network. Experimental hardware environment: Intel i7 3.4G CPU, 8G RAM, GeForce GTX Titan X 8GB GPU. Programming language: python, the framework for deep learning: tensorflow.

In order to be able to train a highly accurate deep learning model, we need a large amount of labeled data. And before that, we first need to establish a standard as a way to label products: qualified and non-qualified products. In fact, in different manufacturing industries, different products are specified with different quality requirements. For example, Table 1 lists some of the gasoline engine carburetor parts of the measurement hole and the size of the nozzle orifice on the carburetor body and its limit deviation (Goodfellow et al., 2014). Within the limit deviation range, it will be marked as qualified product, and beyond the deviation range, it will be marked as non-qualified product.

**Table 1:** Hole size and limit deviation of measuring hole. Lists some of the gasoline engine carburetor parts of the measurement hole and the size of the nozzle orifice on the carburetor body and its limit deviation

Hole size (mm)	Limit deviation (mm)
>0.20∼0.50	±0.008
>0.50∼0.80	±0.010
>0.80∼1.00	±0.012
>1.00∼1.50	±0.015
>1.50∼2.50	±0.020

According to industry standards, we will label different products differently. In the traditional manufacturing industry, the pass or yield rate of a product is obtained by random sampling and inspection. Those products that are randomly selected for testing will be labeled with different quality levels by professionals. And these sampled products will be used as our training data for this experiment, and the tagged grades will be used as labels for the training data. In other words, we do not need to spend extra overhead to generate training data, all the training data are readily available.

### Evaluation criteria

#### (1) Intersection over Union

In addition to measuring our deep learning algorithms through industry standards as a metric, we also adopted Intersection over Union (IoU)as another evaluation metric. IoU is an evaluation metric for common object detection and is used to measure the accuracy of object detectors on a specific dataset. The exact formula for IoU is as follows:

(19) \begin{eqnarray*}IoU= \frac{Area~of~Overlap}{Area~of~Union} .\end{eqnarray*}

#### (2) F-Score

In order to evaluate the accuracy of the algorithm for product quality monitoring and to compare the predicted grade of the algorithm with the actual grade of the product, the ROC indicator is used here for analysis:

True Positive (TP): The true value is qualified, and the predicted value is also qualified;

False Positive (FP): The real value is unqualified, the predicted value is qualified;

True Negative (TN): The real value is qualified, the predicted value is unqualified;

False Negative (FN): The true value is unqualified, and the predicted value is also unqualified.

According to these four indicators, Precision can be calculated, and the predicted result is the probability that the result is qualified and the actual is also qualified:

(20) \begin{eqnarray*}Precision= \frac{TP}{TP+FP} .\end{eqnarray*}

Like Recall, it indicates the probability that the true value of the qualified product is accurately predicted:

(21) \begin{eqnarray*}Recall= \frac{TP}{TP+FN} .\end{eqnarray*}

The corresponding F-Score calculation formula is defined as:

(22) \begin{eqnarray*}F= \frac{2\times Precision\times Recall}{Precision+Recall} .\end{eqnarray*}

#### (3) ROC curve

The Receiver Operating Characteristic(ROC) is a graph comparing the signal (True Positive Rate) to the noise (False Positive Rate). The performance of the model is determined by looking at the Area Under the ROC Curve (AUC). The model works best when the AUC is equal to 1, while an AUC equal to 0.5 indicates the worst model. And any value less than 0.5 means that we perform the exact opposite of what the model suggests. In this case, the True Positive Rate (TPR) can be expressed as:

(23) \begin{eqnarray*}TPR= \frac{TP}{TP+FN} .\end{eqnarray*}

False Positive Rate(FPR)can be expressed as:

(24) \begin{eqnarray*}FPR= \frac{FP}{TN+FP} .\end{eqnarray*}

### Analysis of the results of 3D visualization model construction

#### PASCAL VOC 2012

First, we will use PASCAL VOC 2012 dataset to validate our model. the PASCAL VOC 2012 (Salimans et al., 2016) contains objects in 10 rigid object categories. We will compare Kar et al., 3D-LSTM-1, 3D-GRU-1, 3D-LSTM-3, 3D-GRU-3, Res3D-GRU-3 with our algorithm MapGANs.

The core of Kar et al. (Isola et al., 2017) is to learn a deformable 3D model from 2D annotations in an existing object detection dataset. The model can be driven by noisy automatic object segmentation and complemented by bottom-up modules to recover high frequency shape details. 3D-LSTM-1, 3D-GRU-1, 3D-LSTM-3, 3D-GRU-3 and Res3D-GRU-3 (Ji et al., 2019) are neural networks that learn to generate by mapping from an object image to its underlying 3D shape.

**Figure 3:**
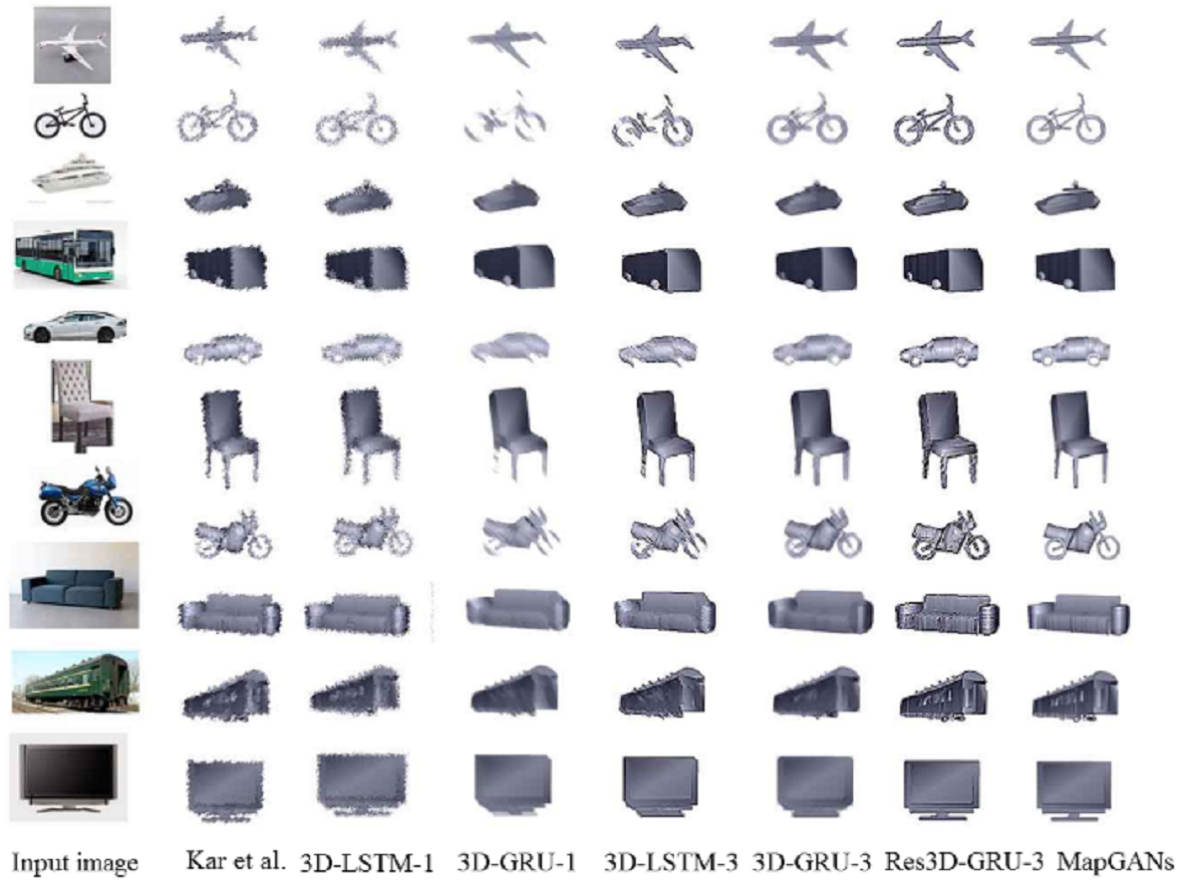
3D renderings constructed by different methods.

Table 2 shows the results of the MapGANs algorithm compared with the other five algorithms. Overall, the Kar et al. (Isola et al., 2017) algorithm is the least effective, the 3D-LSTM-1 and Res3D-GRU-3 algorithms are the secondary, while our MapGANs algorithm obtains the highest accuracy for 6 out of 10 objects. Also, we can see a trend that for some more diverse objects, such as chairs and sofas, all the algorithms cannot achieve a high accuracy rate. In contrast, those with fixed styles, such as cars buses, can be easily recognized by the algorithms. As shown in Fig. 3, the MapGANs algorithm proposed has a good reconstruction effect, The generated object has high resolution on edges and partial details.

**Table 2:** Comparison of Intersection over Union (IoU) results on the PASCAL VOC 2012 dataset. Shows the results of the MapGANs algorithm compared with the other five algorithms.

	Kar et al.	3D-LSTM-1	3D-GRU-1	3D-LSTM-3	3D-GRU-3	Res3D-GRU-3	MapGAN
**Aero**	0.30 ± 0.01	0.47 ± 0.02	0.40 ± 0.02	0.50 ± 0.01	0.45 ± 0.03	0.54 ± 0.01	**0.55 ± 0.01**
**Bike**	0.14 ± 0.02	0.33 ± 0.01	0.35 ± 0.01	0.40 ± 0.04	0.42 ± 0.02	**0.50 ± 0.05**	**0.50 ± 0.03**
**Boat**	0.19 ± 0.05	0.47 ± 0.03	0.47 ± 0.04	0.51 ± 0.01	0.50 ± 0.01	0.56 ± 0.03	**0.61 ± 0.02**
**Bus**	0.50 ± 0.02	0.68 ± 0.03	0.65 ± 0.05	0.73 ± 0.04	0.69 ± 0.06	0.82c0.01	**0.83 ± 0.04**
**Car**	0.47 ± 0.03	0.58 ± 0.05	0.67 ± 0.03	0.62 ± 0.03	**0.71 ± 0.04**	0.70 ± 0.05	0.70 ± 0.02
**Chair**	0.23 ± 0.01	0.20 ± 0.03	0.24 ± 0.04	0.23 ± 0.02	0.28 ± 0.01	0.28 ± 0.01	**0.30 ± 0.03**
**Mbike**	0.36 ± 0.02	0.47 ± 0.06	0.39 ± 0.02	0.63 ± 0.01	**0.66 ± 0.05**	0.65 ± 0.03	0.65 ± 0.04
**Sofa**	0.15 ± 0.04	0.25 ± 0.02	0.31 ± 0.06	0.30 ± 0.06	0.32 ± 0.02	**0.33 ± 0.04**	0.30 ± 0.01
**Train**	0.25 ± 0.05	0.52 ± 0.01	0.61 ± 0.04	0.60 ± 0.03	0.60 ± 0.05	**0.67 ± 0.03**	0.65 ± 0.03
**TV**	0.49 ± 0.03	0.44 ± 0.01	0.35 ± 0.03	0.40 ± 0.07	0.45 ± 0.01	0.57 ± 0.02	**0.58 ± 0.01**

It is worth noting that the PASCAL VOC 2012 dataset only provides images taken from a single angle, so it does not give a good representation of the superiority of our algorithm.

#### Experimental analysis of parts quality inspection

Next we will verify whether MapGANs algorithm can effectively and accurately detect the quality problems of products and parts. Based on the 3D visualization model generated by MapGANs algorithm, the product information, its dimensions, size and shape, etc. will be automatically output. This information will be used to automatically rate the quality level of this product. In this paper, we will discuss only two quality grades: qualified and unqualified. Ultimately, we will compare the quality grades generated by the algorithm with the quality grades assigned to the physical objects by professionals to calculate the accuracy of MapGANs algorithm for quality detection judgments.

In this experiment, we will conduct experiments for seven real workshop parts. All parts are numbered from LJ01 to LJ07, where LJ01 to LJ04 are relatively simple parts, and LJ05 to LJ07 are more complex parts. For each part, we repeated the test 1000 times to minimize the test error.

#### (1) Comparison of quality monitoring accuracy

As mentioned in section 3.1, we will collect a large number of images of the product from different angles and different locations, *i.e.,* two-dimensional images. Then, what will be the result if we perform product quality monitoring by analyzing 2D images only. In this section, we will compare MapGAN and 2D image based methods, as well as 3D-GRU-3 and Res3D-GRU-3.

We build a 2D Convolutional Neural Networks (2D-CNNs) based on 2D images to analyze the taken photos. 2D-CNNs will also generate different parameter metrics about the product and, based on the analyzed metrics, will give a prediction of whether the product quality is up to standard. Meanwhile, since 3D-GRU-3 and Res3D-GRU-3 cannot process multiple images taken from multiple angles at the same time, these two algorithms will process multiple images of the same part separately, and finally take their average as the final prediction result.

**Figure 4:**
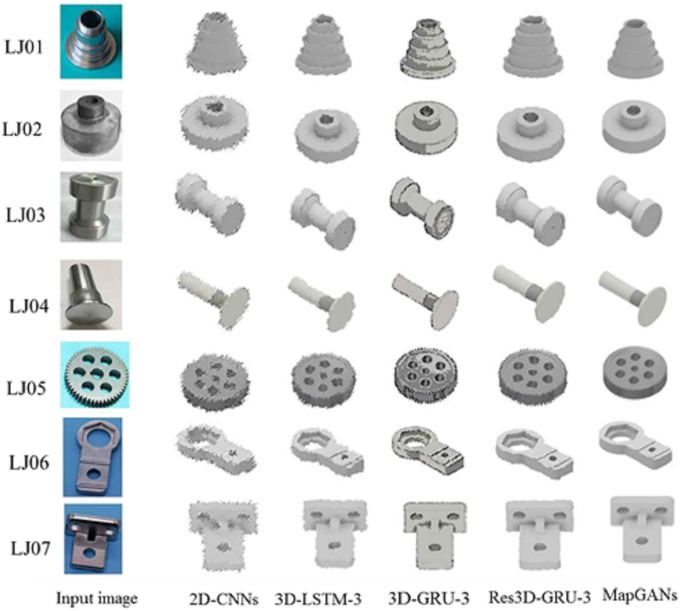
3D model effect of parts LJ01 to LJ07.

Figure 4 shows the reconstruction effect of LJ01 to LJ07 using MapGANs, 3D-LSTm-3,3D-GRU-3, RES3D-GRU-3 and 2D-CNNS. MapGANs worked better than several other methods. In particular, it has better accuracy in the details.

**Figure 5:**
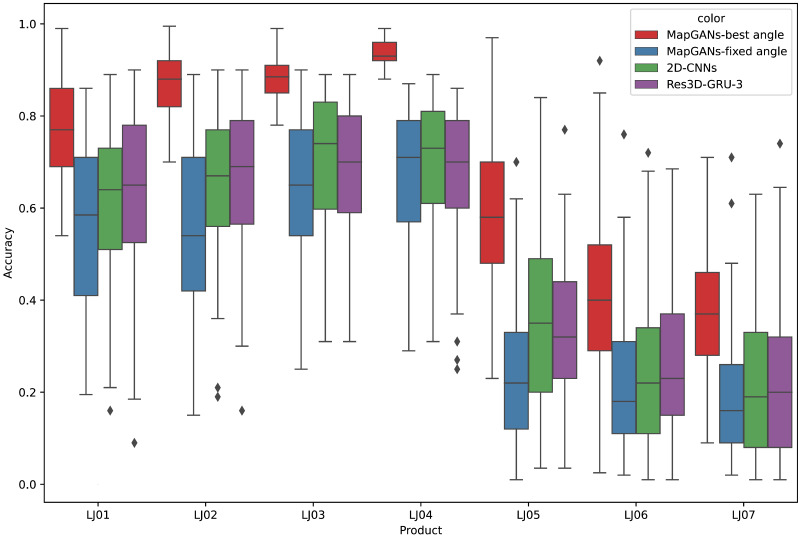
Comparison of quality monitoring accuracy.

Figure 5 shows the comparison of MapGANs, 3D-GRU-3, Res3D-GRU-3 and 2D-CNNs in terms of quality monitoring accuracy. The results show that the performance of MapGANs is far better than several other methods. Especially on some complex components (LJ05, LJ06 and LJ07), the accuracy of the other three algorithms is very low, with 2D-CNNs performing the worst. On the contrary, MapGANs can well determine whether the product quality meets the standard. The main reason, in two-dimensional images, it is difficult to accurately judge the parameter index of products from a single angle because of the angle of shooting and the shape of products, while our MapGANs can clearly show the size, dimensions and shapes of products in different dimensions and different angles. Therefore, MapGANs can achieve a very high accuracy rate of quality detection.

#### (2) F-Score indicators for MapGANs

We also computed the metrics of Precision, Recall and F-Score for MapGANs. Table 3 lists the specific experimental results. We can see that MapGANs achieve very high prediction correctness (average F-Score of 0.87 on relatively simple parts). In particular, on the three parts LJ02, LJ03, and LJ04, the accuracy is higher than 0.90. While on relatively more complex parts, the accuracy of MapGANs decreases.

**Figure 6:**
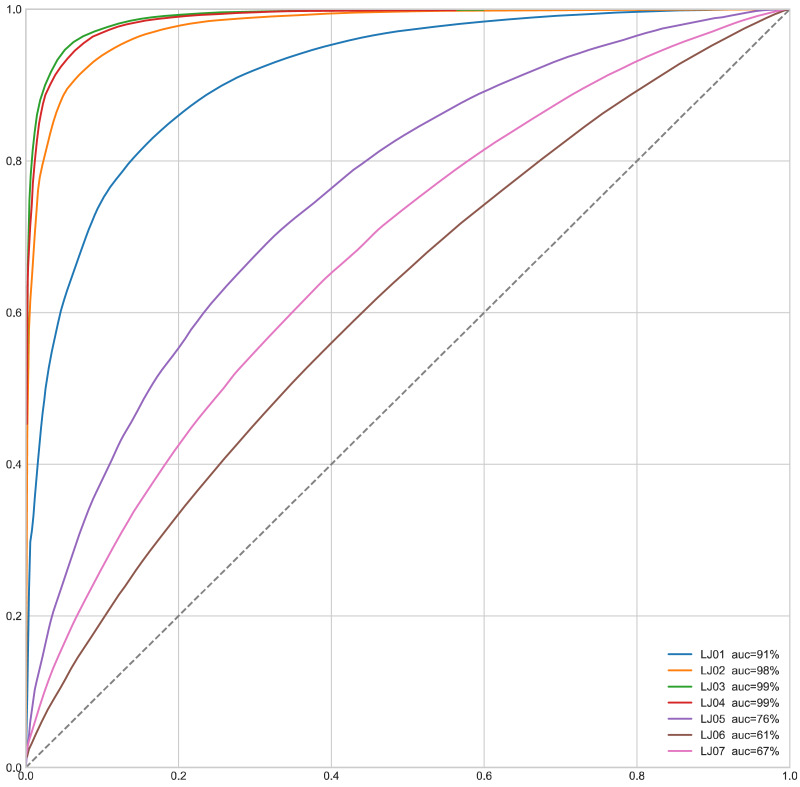
ROC curve of MapGANs.

We also draw the ROC curves of MapGANs to compare True Positive Rate and False Positive Rate. the dashed line in Fig. 6 indicates the random value, which is the worst case of the model. And the steeper curve means that the model is better. We can see that the result is very consistent with the results of the F-Score in Table 3. Especially on the first four parts, MapGANs works best with the highest accuracy, while the effect drops significantly on the last three more complex parts. However, in general, MapGANs can achieve very high accuracy to automatically detect the quality of product components.

### Analysis of different installation methods for cameras

The camera installation method plays a crucial role in generating 3D visualization models. In this section, we discuss the impact of different camera setup methods on the generation of 3D visualization models by MapGANs.

All the cameras here are installed under the guidance of professionals to ensure that each camera is shot from the best angle. At the same time, for different parts, the camera’s shooting angle will be adjusted accordingly. We tested the accuracy of MapGANs for the 2, 4, 6 and 8 camera cases, respectively. Table 4 lists the detailed testing results. The results show that MapGANs can achieve over 80% accuracy with six cameras, and 85% with 8 cameras. Specifically, for some simpler parts, four cameras can already achieve a high accuracy rate. For some more complex parts, more cameras are needed so that MapGANs can fill in the gaps in the 3D model due to occlusion and other factors based on multiple 2D images, and finally generate a more accurate 3D visualization model.

**Table 3:** Quality detection results of MapGANs algorithm. Lists the specific experimental results.

	Precision	Recall	*F*-Score
**LJ01**	0.82	0.73	0.77
**LJ02**	0.90	0.92	0.91
**LJ03**	0.88	0.95	0.91
**LJ04**	0.98	0.94	0.96
**LJ05**	0.61	0.54	0.57
**LJ06**	0.45	0.38	0.41
**LJ07**	0.47	0.40	0.43
average	**0.73**	**0.70**	**0.71**

**Table 4:** *F*-Score of MapGANs algorithm for different number of cameras. Lists the detailed testing results.

Number of cameras	2	4	6	8
** LJ01**	0.60	0.67	0.73	0.77
**LJ02**	0.74	0.84	0.90	0.91
** LJ03**	0.78	0.85	0.91	0.91
**LJ04**	0.80	0.90	0.96	0.96
** LJ05**	0.33	0.46	0.52	0.57
**LJ06**	0.30	0.31	0.35	0.41
** LJ07**	0.27	0.32	0.35	0.43
average	**0.55**	**0.62**	**0.67**	**0.71**

For different parts, professionals adjust the position of the cameras to ensure that all cameras are shooting at the best angle. To confirm whether the camera position affects the quality inspection results, we also conducted another set of experiments. In this experiment, we fixed the camera angle so that the 8 cameras were evenly distributed around the part.

**Figure 7:**
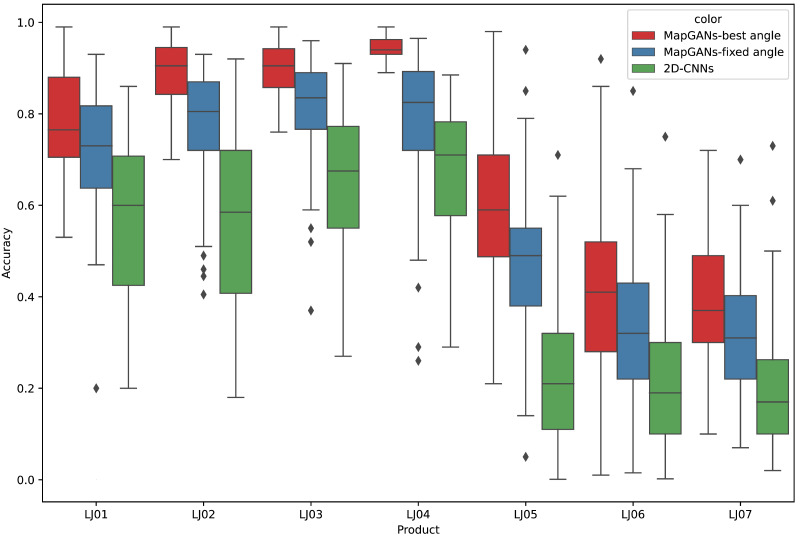
The effect of different shooting angles.

The experimental results are shown in Fig. 7. MapGANs-Best Angle refers to the MapGANs algorithm at the best shooting angle, MapGANs-Fixed Angle refers to the fixed shooting angle, and 2D-CNNs refers to the use of CNN algorithm on a single 2D image. We can see that the difference of shooting angle will affect the final prediction result to some extent. The prediction accuracy of the MapGANs algorithm for the fixed shooting angle case is slightly lower than that of the best angle shooting result. The main reason is that the MapGANs algorithm can use images taken at different angles to make associations and complements in order to minimize the influence of factors such as camera occlusion. For some more complex models, the best shooting angle can obtain better detection accuracy. Moreover, MapGANs far outperforms 2D-CNNs for images taken at fixed angles or not.

## Conclusion

This paper discusses how computer vision techniques can be applied to automatic component production quality monitoring. Based on this, a new generative adversarial network, MapGANs, is proposed in this paper to automatically generate 3D visualization models from 2D views taken from multiple angles and locations. Then, each parameter index of the product is judged by the generated 3D visualization model. The experiment shows when the image is taken from a single Angle, MapGANs is still better than other methods, although the overall performance of MapGANs is not good. The accuracy of MapGANs method is superior to other methods for 3D reconstruction in six and eight cameras. It can restore the details of the parts well and achieve a better detection accuracy. Since MapGANs uses multi-discrimination and multi-generator for adversarial training, 3D reconstruction of objects photographed from multiple angles has a good effect.

## Supplemental Information

10.7717/peerj-cs.768/supp-1 Supplemental Information 1 Dataset of furniture Industrial parts data is not given due to commercial reasons. We added some furniture data for testing.

10.7717/peerj-cs.768/supp-2 Supplemental Information 2 Code of Multi-angle projective Generative Adversarial Networks The major code, which implements Multi-angle projective Generative Adversarial Networks (MapGANs), to automatically generate 3D visualization models of products and components.

## Additional Information and Declarations

### Data Availability

The following information was supplied regarding data availability: The code and dataset are available in the Supplementary Files.
