# Supplementary figures and images for: 3D visualization model construction based on generative adversarial networks
Source: PeerJ Comput Sci. 2022 Mar 29;8:e768. doi: 10.7717/peerj-cs.768 (PMC9044199; doi:10.7717/peerj-cs.768)

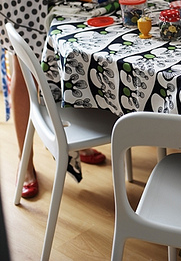

Supplement: Supplemental Information 1 — Industrial parts data is not given due to commercial reasons. We added some furniture data for testing. [file peerj-cs-08-768-s001.zip › image_samples/0071.png]

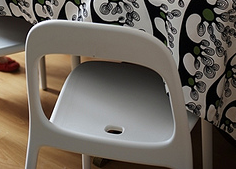

Supplement: Supplemental Information 1 — Industrial parts data is not given due to commercial reasons. We added some furniture data for testing. [file peerj-cs-08-768-s001.zip › image_samples/0070.png]

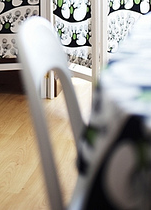

Supplement: Supplemental Information 1 — Industrial parts data is not given due to commercial reasons. We added some furniture data for testing. [file peerj-cs-08-768-s001.zip › image_samples/0072.png]

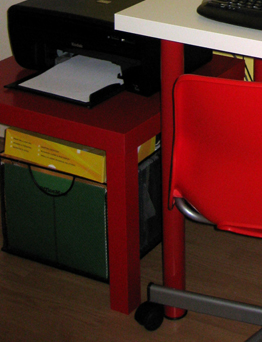

Supplement: Supplemental Information 1 — Industrial parts data is not given due to commercial reasons. We added some furniture data for testing. [file peerj-cs-08-768-s001.zip › image_samples/0112.png]

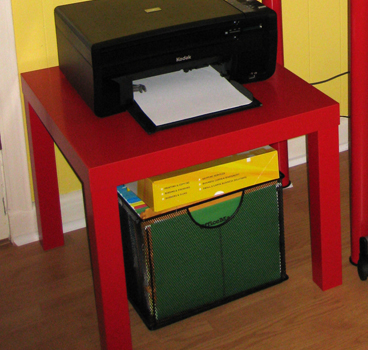

Supplement: Supplemental Information 1 — Industrial parts data is not given due to commercial reasons. We added some furniture data for testing. [file peerj-cs-08-768-s001.zip › image_samples/0113.png]

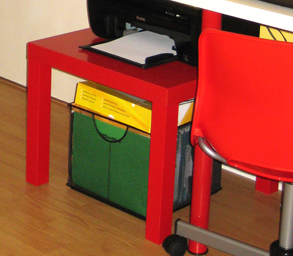

Supplement: Supplemental Information 1 — Industrial parts data is not given due to commercial reasons. We added some furniture data for testing. [file peerj-cs-08-768-s001.zip › image_samples/0114.png]

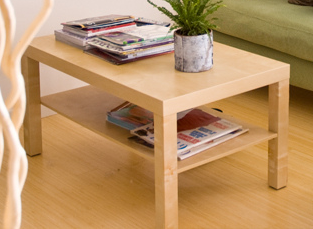

Supplement: Supplemental Information 1 — Industrial parts data is not given due to commercial reasons. We added some furniture data for testing. [file peerj-cs-08-768-s001.zip › image_samples/0159.png]

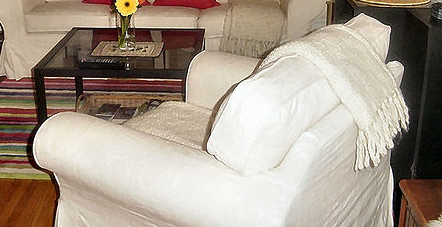

Supplement: Supplemental Information 1 — Industrial parts data is not given due to commercial reasons. We added some furniture data for testing. [file peerj-cs-08-768-s001.zip › image_samples/0164.png]

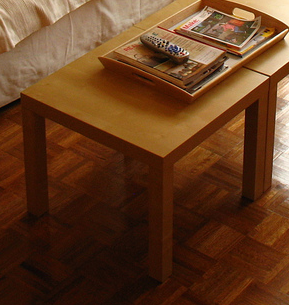

Supplement: Supplemental Information 1 — Industrial parts data is not given due to commercial reasons. We added some furniture data for testing. [file peerj-cs-08-768-s001.zip › image_samples/0004.png]

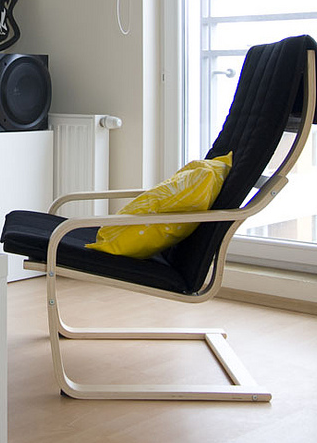

Supplement: Supplemental Information 1 — Industrial parts data is not given due to commercial reasons. We added some furniture data for testing. [file peerj-cs-08-768-s001.zip › image_samples/0148.png]

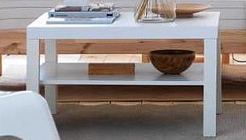

Supplement: Supplemental Information 1 — Industrial parts data is not given due to commercial reasons. We added some furniture data for testing. [file peerj-cs-08-768-s001.zip › image_samples/0160.png]

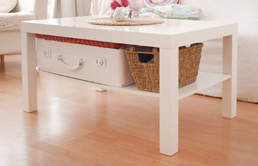

Supplement: Supplemental Information 1 — Industrial parts data is not given due to commercial reasons. We added some furniture data for testing. [file peerj-cs-08-768-s001.zip › image_samples/0161.png]

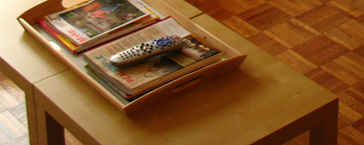

Supplement: Supplemental Information 1 — Industrial parts data is not given due to commercial reasons. We added some furniture data for testing. [file peerj-cs-08-768-s001.zip › image_samples/0003.png]

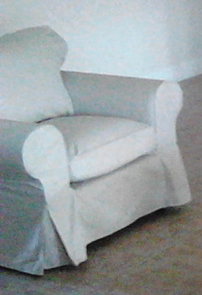

Supplement: Supplemental Information 1 — Industrial parts data is not given due to commercial reasons. We added some furniture data for testing. [file peerj-cs-08-768-s001.zip › image_samples/0163.png]

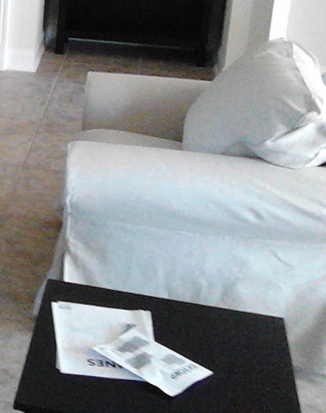

Supplement: Supplemental Information 1 — Industrial parts data is not given due to commercial reasons. We added some furniture data for testing. [file peerj-cs-08-768-s001.zip › image_samples/0162.png]

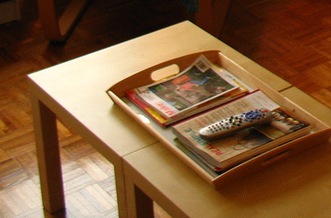

Supplement: Supplemental Information 1 — Industrial parts data is not given due to commercial reasons. We added some furniture data for testing. [file peerj-cs-08-768-s001.zip › image_samples/0002.png]

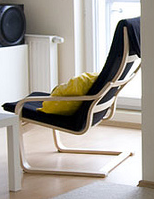

Supplement: Supplemental Information 1 — Industrial parts data is not given due to commercial reasons. We added some furniture data for testing. [file peerj-cs-08-768-s001.zip › image_samples/0147.png]

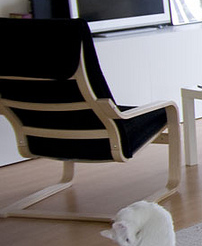

Supplement: Supplemental Information 1 — Industrial parts data is not given due to commercial reasons. We added some furniture data for testing. [file peerj-cs-08-768-s001.zip › image_samples/0146.png]

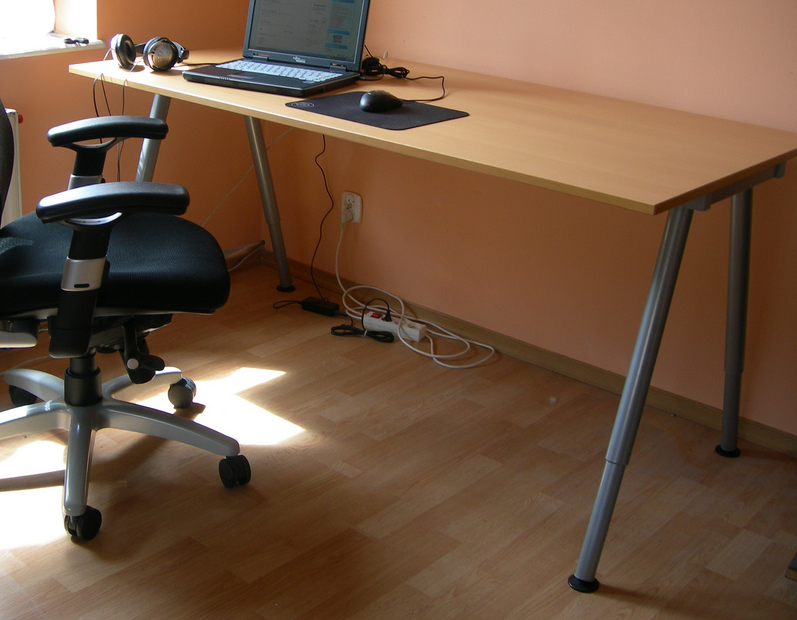

Supplement: Supplemental Information 1 — Industrial parts data is not given due to commercial reasons. We added some furniture data for testing. [file peerj-cs-08-768-s001.zip › image_samples/00002.png]

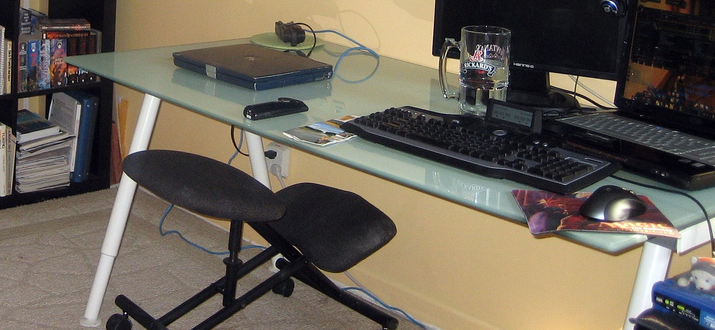

Supplement: Supplemental Information 1 — Industrial parts data is not given due to commercial reasons. We added some furniture data for testing. [file peerj-cs-08-768-s001.zip › image_samples/00003.png]

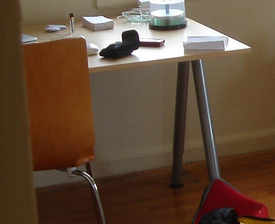

Supplement: Supplemental Information 1 — Industrial parts data is not given due to commercial reasons. We added some furniture data for testing. [file peerj-cs-08-768-s001.zip › image_samples/00001.png]

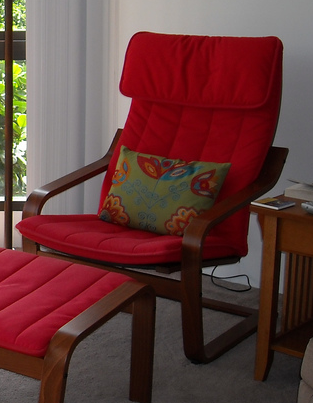

Supplement: Supplemental Information 1 — Industrial parts data is not given due to commercial reasons. We added some furniture data for testing. [file peerj-cs-08-768-s001.zip › image_samples/0044.png]

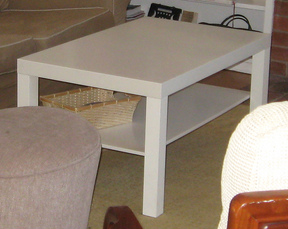

Supplement: Supplemental Information 1 — Industrial parts data is not given due to commercial reasons. We added some furniture data for testing. [file peerj-cs-08-768-s001.zip › image_samples/0051.png]

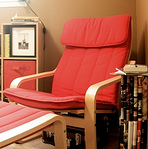

Supplement: Supplemental Information 1 — Industrial parts data is not given due to commercial reasons. We added some furniture data for testing. [file peerj-cs-08-768-s001.zip › image_samples/0045.png]

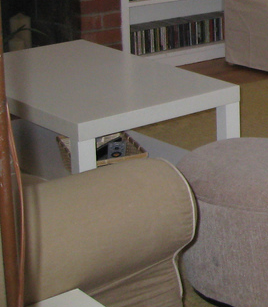

Supplement: Supplemental Information 1 — Industrial parts data is not given due to commercial reasons. We added some furniture data for testing. [file peerj-cs-08-768-s001.zip › image_samples/0053.png]

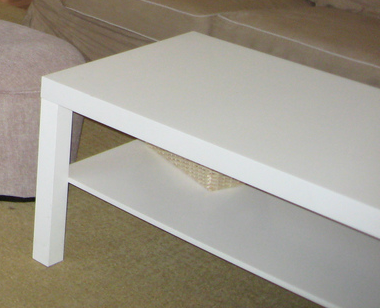

Supplement: Supplemental Information 1 — Industrial parts data is not given due to commercial reasons. We added some furniture data for testing. [file peerj-cs-08-768-s001.zip › image_samples/0052.png]

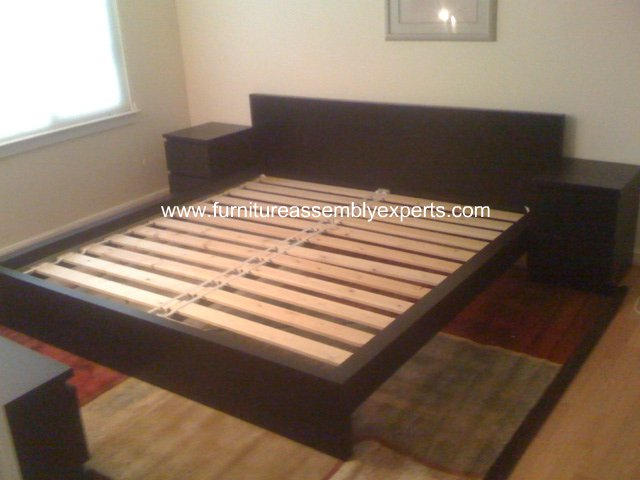

Supplement: Supplemental Information 1 — Industrial parts data is not given due to commercial reasons. We added some furniture data for testing. [file peerj-cs-08-768-s001.zip › image_samples/0056.png]

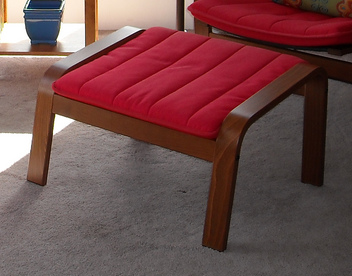

Supplement: Supplemental Information 1 — Industrial parts data is not given due to commercial reasons. We added some furniture data for testing. [file peerj-cs-08-768-s001.zip › image_samples/0043.png]

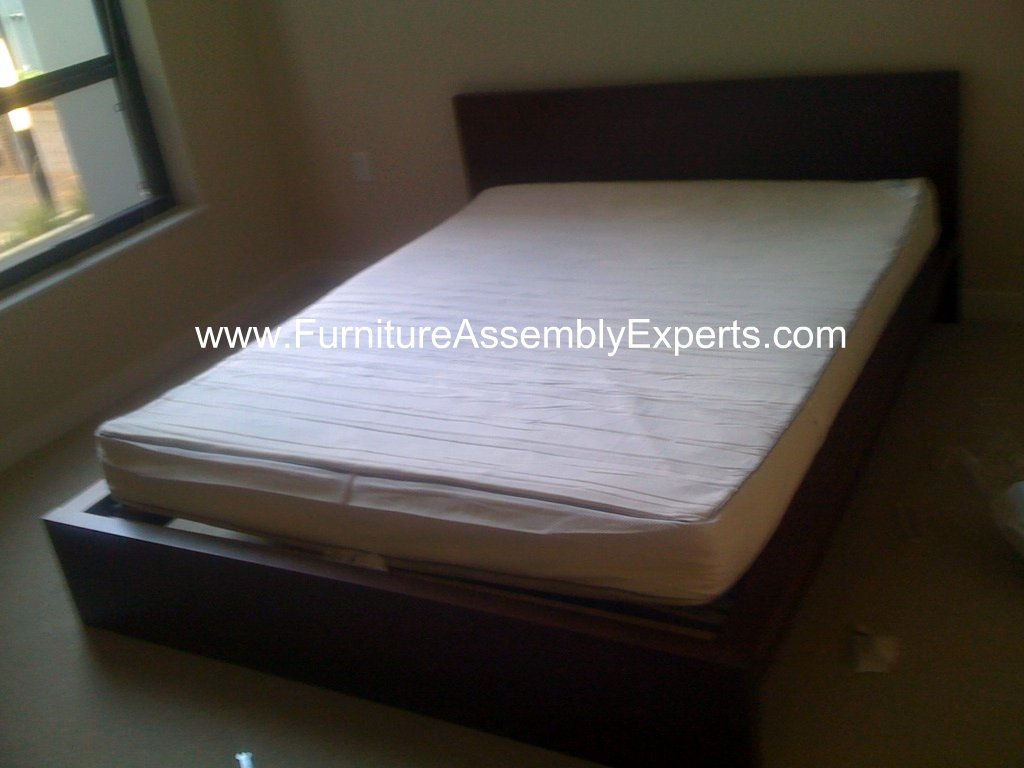

Supplement: Supplemental Information 1 — Industrial parts data is not given due to commercial reasons. We added some furniture data for testing. [file peerj-cs-08-768-s001.zip › image_samples/0057.png]

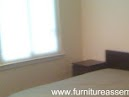

Supplement: Supplemental Information 1 — Industrial parts data is not given due to commercial reasons. We added some furniture data for testing. [file peerj-cs-08-768-s001.zip › image_samples/0055.png]
